# Notice

**Published:** 1875-08

**Authors:** 


### Notice.

We have sent out our bills for our yearly subscriptions, and we take this opportunity to remind those of our subscribers yet in arrears. Changes have been made in the financial department of the JOURNAL, and we wish those who have not remitted would do so at once.

Remittances should be made by draft or postoffice order, or by registered letter. To save trouble, please make them payable to Dr. E. N. Brush. Direct all communications to No. 8 South Division street when intended for the JOURNAL. Dr. J. F. Miner's address is 978 Main street where private letters should be addressed. Some complaint has been made that the JOURNAL has failed to reach subscribers, we would thank any who do not receive their copies regularly to inform us, and shall be happy to furnish back numbers to those who have failed to receive them.

